# Spontaneous Rupture of an Unscarred Gravid Uterus in a Primigravid Woman at 32 Weeks of Gestation

**DOI:** 10.1155/2014/209585

**Published:** 2014-06-30

**Authors:** Etsuko Mizutamari, Tomoko Honda, Takashi Ohba, Hidetaka Katabuchi

**Affiliations:** Department of Obstetrics and Gynecology, Faculty of Life Science, Kumamoto University, 1-1-1 Honjo, Chuo-ku, Kumamoto 860-8556, Japan

## Abstract

Uterine rupture usually occurs in a scarred uterus, especially secondary to prior cesarean section. Antepartum uterine rupture in an unscarred uterus is extremely rare. We report a case of spontaneous rupture of an unscarred gravid uterus at 32 weeks of gestation in a primigravid woman. Ultrasonography and magnetic resonance imaging showed a bulging cystic lesion communicating with the intrauterine cavity. Operative findings during emergent cesarean section revealed uterine perforation in the right cornual area and a prolapsed, nonbleeding amniotic sac. The left cornual area was also focally thin. An arcuate uterus was suspected based on follow-up hysterosalpingography. Antepartum uterine rupture tends to occur in the uterine cornual area. In this case, Müllerian duct anomalies may have been associated with focal myometrial defects.

## 1. Introduction

Uterine rupture is an obstetric complication that causes significant maternal and fetal morbidity and mortality. It is associated with prolonged labor, oxytocin induction, and uterine scarring, especially following prior cesarean section [1]. Several reports have indicated that antepartum uterine rupture can occur in an unscarred uterus, but the contributory risk factors have not been identified [2–7].

Here we report a case of spontaneous rupture of an unscarred gravid uterus with favorable fetal outcome at 32 weeks of gestation.

## 2. Case Presentation

A healthy 29-year-old primigravid woman conceived naturally and began receiving prenatal checkups at a private hospital. At week 31, fetal bilateral hydronephrosis was observed, and the subject was referred to Kumamoto University Hospital at 31 weeks + 6 days of gestation for diagnostic studies and proper management. She had no history of trauma, uterine surgery, or intrauterine intervention, or symptoms suggesting the presence of Ehlers-Danlos syndrome in either herself or her family members.

Abdominal ultrasonography revealed bilateral hydronephrosis and a small ventricular septal defect in the fetus, but no structural abnormalities of the uterus, though no special attention was paid to the uterine wall. Four days later, the subject suffered from acute right upper quadrant abdominal pain and visited a private hospital. Abdominal ultrasonography revealed an anechoic cystic lesion in the region of her symptoms. A cardiotocogram showed a reassuring fetal heart late pattern with periodical uterine contractions. A drip infusion of ritodrine hydrochloride was administered and the subject was immediately transferred to Kumamoto University Hospital at 32 weeks + 3 days of gestation.

On admission, she was not in acute distress. Her abdomen was soft, and a fist-sized elastic soft mass was palpated at the upper right quadrant. Speculum examination revealed a normally positioned cervix and no vaginal bleeding. On digital examination the* cervix* was tightly closed and uneffaced. Her vital signs were stable, and she had a hemoglobin level of 12.2 g/dL. A cardiotocogram showed fetal tachycardia of 180 bpm with moderate baseline variability and nonperiodic accelerations. Periodic uterine contractions occurred every 6–8 min.

Abdominal ultrasonography demonstrated a defect in the uterine wall at the right side near the uterine fundus. The amniotic cavity was bulging out of the uterus, corresponding to the palpable mass. The placenta was situated in the fundus and there was no evidence of placental abruption. There was a normal amount of amniotic fluid.

Though ultrasonographic findings led us to speculate uterine rupture, her clinical course did not suggest such a life threatening complication. So she underwent magnetic resonance imaging (MRI) examination to check uterine anomalies.

**Figure 1:**
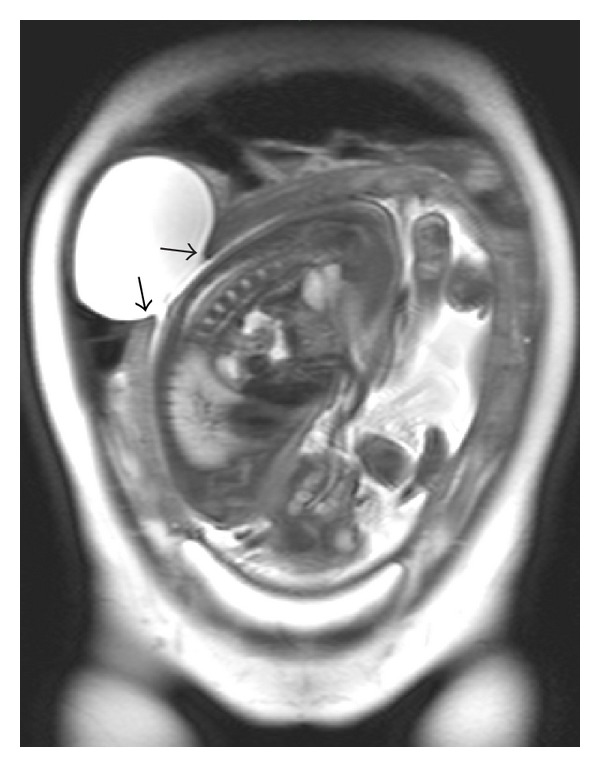
Magnetic resonance imaging (T2WI, coronal section) of the pelvis revealed a bulging amniotic cavity protruding through the defect in the uterine wall (arrows). Neither peritoneal fluid nor hemoperitoneum was observed.

MRI confirmed an 8.5-cm diameter extrauterine cyst protruding from the intrauterine amniotic cavity (Figure 1). These findings led us to suspect the possibility of an impending uterine rupture, and we, therefore, performed an emergent cesarean section.

**Figure 2:**
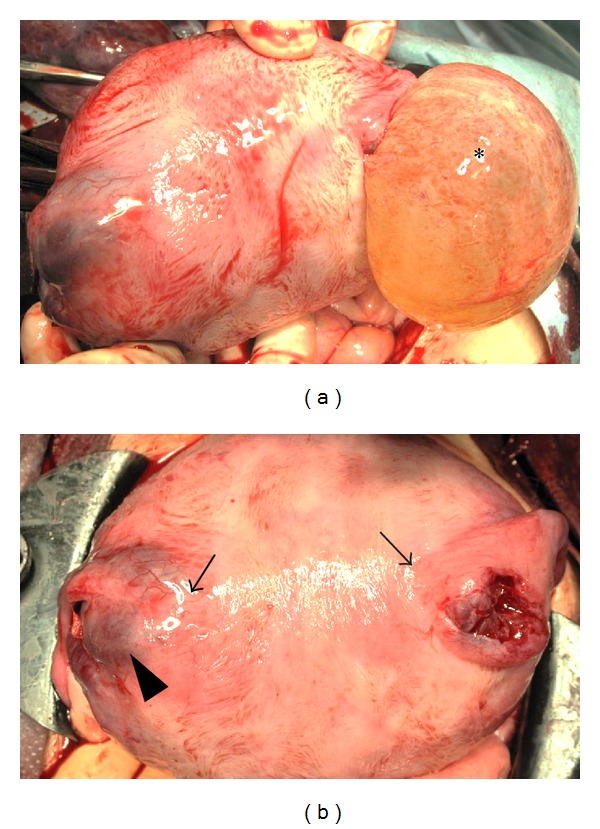
(a) Macroscopic appearance of the uterine fundus before placenta removal. A uterine perforation was located at the right corneal area, and the amniotic sac (∗) was prolapsed. (b) Macroscopic appearance of the uterine fundus after placenta removal. The uterine perforation was focal and located just behind the cornual end of the right fallopian tube. The left cornual area (arrowhead) was focally thin, and the cornual ends of both fallopian tubes (arrows) seemed closer to the midline of uterus than normal.

No peritoneal fluid or hemoperitoneum was detected, and the anterior surface of the uterus had a normal appearance. A lower segment cesarean section was performed, and the subject gave birth to a male infant weighing 1,788 g with Apgar scores of 6 at 1 min and 9 at 5 min. The amniotic fluid was clear. A 2-cm diameter uterine perforation was located at the right cornual area, with prolapse of the amniotic sac (Figure 2(a)). There was no bleeding at the perforation. On uterine examination, the perforation was located behind the cornual end of right fallopian tube. The left side of the cornual area was also focally thin, and the cornual ends of both fallopian tubes seemed closer to the midline of the uterus than normal (Figure 2(b)). The cornual areas were repaired with interrupted vicryl sutures. The total blood loss was approximately 500 g without blood transfusion.

**Figure 3:**
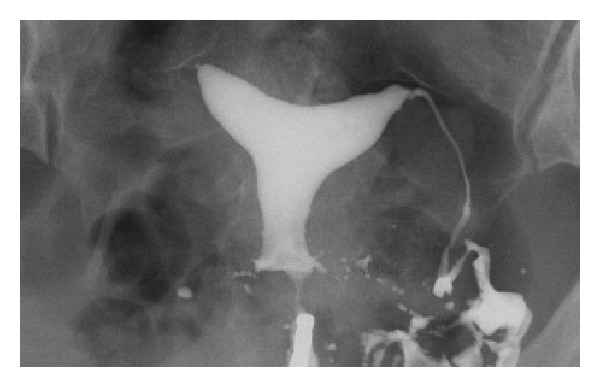
Hysterosalpingography at 8 months after cesarean section revealed mild indentation of the endometrium at the uterine fundus with right tubal occlusion.

The subject's recovery period was uneventful, and she was discharged on the eighth postoperative day. MRI examination performed 4 months after the cesarean section revealed no uterine deformity, and follow-up hysterosalpingography (HSG) at 8 months showed an arcuate uterus with right tubal occlusion (Figure 3).

The infant was diagnosed with bilateral hydronephrosis and ventricular septal defect but did not require surgery and remained in stable condition and was discharged 10 weeks after birth.

## 3. Discussion

Rupture of the gravid uterus is an emergency condition causing high fetomaternal mortality and morbidity. In developed countries the prevalence of uterine rupture in pregnant women with previous cesarean section has been reported to be approximately 1%, whereas it is extremely rare in women without a history of cesarean section [8]. Several risk factors may contribute to uterine rupture of the gravid uterus in women with no history of uterine surgery, including intrauterine surgery, multiparity, oxytocin stimulation, placenta accreta, Ehlers-Danlos syndrome, cocaine abuse, in-utero exposure to diethylstilbestrol, uterine anomalies, and obstructed labor, for instance, due to undiagnosed fetopelvic disproportion or malpresentation [1, 7, 9–14]. Because the key factor in uterine rupture during pregnancy is the contraction of the uterine musculature due to labor, careful intrapartum monitoring of uterine activity can lead to the correct diagnosis.

In the English literature there are six reported cases of antepartum uterine rupture of the unscarred uterus in the absence of any identified risk factors [2–7] (Table 1). Half of these cases (3/6) were primigravid women. All cases were associated with symptoms of acute abdomen with prominent hemoperitoneum, occurring primarily in the third trimester. Two-thirds (4/6) of fetuses died before delivery; however, all patients recovered following treatment. The most common rupture sites were the cornual area and the uterine fundus.

In addition to these six cases with acute abdomen, there were two interesting cases of antepartum “silent” uterine rupture whose clinical findings were similar to those in this report [15, 16]. First, the patients were in no acute distress and had no hemoperitoneum. Second, uterine perforations were located at the cornual area. Third, focal loss of myometrial thickness was detected in the counterpart of the ruptured cornual area. In our case, follow-up HSG revealed that the subject had an arcuate uterus, a common congenital uterine anomaly affecting 3.9% of all women [17]. We are not aware of any reports suggesting a correlation between spontaneous uterine rupture and arcuate uterus. Though we cannot definitively identify the mechanisms involved in this case, based on our findings we speculate that Müllerian duct anomalies may have been related to focal weakness of the bilateral cornual uterine myometrium.

In conclusion, while spontaneous uterine rupture of the unscarred gravid uterus is extremely rare, it usually occurs in the cornual area. Further studies are needed to determine the etiology of cornual myometrial defects and the relation to Müllerian duct anomalies.

## Figures and Tables

**Table 1:** Antepartum uterine rupture in the unscarred uterus with no identified risk factors.

Case	Age	G-P	GA	Initial presentation	Rupture site	Fetal outcome	Reference
1	20	1-0	37	Abdominal pain Loss of FM	Lt. cornual area	Stillbirth	[2]
2	27	1-0	32	Abdominal pain Nausea	Rt. uterosacral area	Live birth	[3]
3	31	1-0	21∗	Abdominal pain	Lt. cornual area	Live birth	[4]
4	26	3-2	32	Abdominal pain Loss of FM	Rt. cornual area~Fundus	Stillbirth	[5]
5	29	2-1	32	Abdominal pain Loose stool	Lower segment~Fundus	Stillbirth	[6]
6	31	3-2	17	Abdominal pain	Fundus	Stillbirth	[7]

G-P; gravida-para, GA; gestational age (weeks), Lt.; left, Rt.; right, FM; fetal movement.

*The rupture site was repaired, and the subject underwent cesarean section at 33 weeks gestation due to premature rupture of the amniotic membranes.
